# The coArtHA trial—identifying the most effective treatment strategies to control arterial hypertension in sub-Saharan Africa: study protocol for a randomized controlled trial

**DOI:** 10.1186/s13063-021-05023-z

**Published:** 2021-01-21

**Authors:** Herry Mapesi, Ravi Gupta, Herieth Ismael Wilson, Blaise Lukau, Alain Amstutz, Aza Lyimo, Josephine Muhairwe, Elizabeth Senkoro, Theonestina Byakuzana, Madavida Mphunyane, Moniek Bresser, Tracy Renée Glass, Mark Lambiris, Günther Fink, Winfrid Gingo, Manuel Battegay, Daniel Henry Paris, Martin Rohacek, Fiona Vanobberghen, Niklaus Daniel Labhardt, Thilo Burkard, Maja Weisser

**Affiliations:** 1 Ifakara Health Institute, Ifakara branch, Ifakara, United Republic of Tanzania; 2 Swiss Tropical and Public Health Institute (Swiss TPH), Basel, Switzerland; 3 University of Basel, Basel, Switzerland; 4 SolidarMed, Partnerships for Health, Maseru, Lesotho; 5 Division of Infectious Diseases and Hospital Epidemiology, University Hospital Basel, Basel, Switzerland; 6 St. Francis Referral Hospital, Ifakara, United Republic of Tanzania; 7 Tanzania Training Center for International Health, Ifakara, United Republic of Tanzania; 8 Ministry of Health, Maseru, Lesotho; 9 Medical Outpatient and Hypertension Clinic, ESH Hypertension Centre of Excellence, University Hospital Basel, Basel, Switzerland; 10 Department of Cardiology, University Hospital Basel, Basel, Switzerland

**Keywords:** Arterial hypertension, Blood pressure, Antihypertensive therapy, Randomized controlled trial, Sub-Saharan Africa, HIV, Triple therapy, Dual therapy, Tanzania and Lesotho

## Abstract

**Background:**

Arterial hypertension is the most prevalent risk factor for cardiovascular disease in sub-Saharan Africa. Only a few and mostly small randomized trials have studied antihypertensive treatments in people of African descent living in sub-Saharan Africa.

**Methods:**

In this open-label, three-arm, parallel randomized controlled trial conducted at two rural hospitals in Lesotho and Tanzania, we compare the efficacy and cost-effectiveness of three antihypertensive treatment strategies among participants aged ≥ 18 years. The study includes patients with untreated uncomplicated arterial hypertension diagnosed by a standardized office blood pressure ≥ 140/90 mmHg. The trial encompasses a superiority comparison between a triple low-dose antihypertensive drug combination versus the current standard of care (monotherapy followed by dual treatment), as well as a non-inferiority comparison for a dual drug combination versus standard of care with optional dose titration after 4 and 8 weeks for participants not reaching the target blood pressure. The sample size is 1268 participants with parallel allocation and a randomization ratio of 2:1:2 for the dual, triple and control arms, respectively. The primary endpoint is the proportion of participants reaching a target blood pressure at 12 weeks of ≤ 130/80 mmHg and ≤ 140/90 mmHg among those aged < 65 years and ≥ 65 years, respectively. Clinical manifestations of end-organ damage and cost-effectiveness at 6 months are secondary endpoints.

**Discussion:**

This trial will help to identify the most effective and cost-effective treatment strategies for uncomplicated arterial hypertension among people of African descent living in rural sub-Saharan Africa and inform future clinical guidelines on antihypertensive management in the region.

**Trial registration:**

Clinicaltrials.govNCT04129840. Registered on 17 October 2019 (https://www.clinicaltrials.gov/).

## Background

Cardiovascular morbidity and mortality in low- and middle-income countries—particularly sub-Saharan Africa—are rising [1, 2]. The most important risk factor for cardiovascular disease in sub-Saharan Africa is arterial hypertension with a prevalence of 30–46% [3–9] and an age-standardized mean systolic blood pressure (BP) being 5–20 mmHg higher compared to North America or Europe [1]. Black ethnicity has been associated with elevated BP [10] due to genetic factors, epigenetic adaptation to climate [11, 12], and increased susceptibility to salt intake [13, 14]. Moreover, complications of arterial hypertension such as stroke, chronic kidney disease, and myocardial infarction have shown to be more prevalent in black compared to white populations [15]. Despite the high burden of arterial hypertension in sub-Saharan Africa, less than 40% of hypertensive patients are aware of their diagnosis. Among those who are aware of their diagnosis, less than 30% are receiving antihypertensive medications and less than 20% of those being treated have a controlled BP [6, 16].

Most patients need a combination of at least two antihypertensive drugs to achieve BP control [17–19]. The latest American and European guidelines recommend starting a combination pharmacologic treatment with at least two classes of antihypertensive medications for patients with a BP ≥ 140/90 mmHg [20]. However, the World Health Organization (WHO) guidelines still recommend a sequential treatment approach starting with a calcium channel blocker (CCB) or a thiazide diuretic (TZD), and combining both drugs only in case of inadequate response [21–23]. From sub-Saharan Africa, there is very little evidence supporting the WHO approach: Only five, mostly small randomized trials comparing the effectiveness of different antihypertensive regimens were conducted in sub-Saharan Africa [24–28]. A recent trial performed in ten centers in six African countries found amlodipine-containing regimens with either hydrochlorothiazide or perindopril to be superior to perindopril plus hydrochlorothiazide in controlling BP at 6 months [29].

The control Arterial Hypertension in sub-Saharan Africa (coArtHA) trial aims at comparing three treatment strategies to achieve rapid BP control with widely available drugs within 12 weeks in participants of African descent in rural sub-Saharan Africa. In addition, it assesses hypertension-mediated organ damage and compares the cost-effectiveness of the three treatment strategies considered.

## Methods

### Study setting

The coArtHA trial is conducted at the St. Francis Referral Hospital in Ifakara, Southwestern Tanzania, and Mokhotlong District Hospital, Mokhotlong town, Northern Lesotho. In Tanzania, the STEP survey 2013 showed a prevalence of arterial hypertension of 25.9% in individuals aged 24–65 years of age [30]. At the Chronic Diseases Clinic of Ifakara (CDCI) of the St. Francis Referral Hospital, participants of the Kilombero and Ulanga Antiretroviral Cohort (KIULARCO) [31, 32] were hypertensive at enrolment in 12% [33]. Among HIV-positive patients on stable ART prevalence of arterial hypertension was even higher with 27% overall and 44% among patients aged ≥ 50 years [34]. The CDCI cares for about 4500 patients with an HIV infection, while the general outpatient department sees 36,000 patients a year [35].

In Lesotho, prevalence of arterial hypertension in the general population is around 31% among persons aged 25 to 64 years [36] and 28% and 22% among HIV-positive females and males, respectively [37]. Mokhotlong Hospital serves the district of Mokhotlong, which is situated in northeast of Lesotho and has about 120,000 habitants, the majority living in remote villages scattered over a mountainous area of 4075 km^2^. The hospital has 110 beds and its outpatient clinic serves 4500–7500 adult patients per month.

### Study design

The coArtHA trial is an investigator-initiated, open-label, three-arm randomized controlled two-country trial to compare the effectiveness and cost-effectiveness of three antihypertensive treatment strategies in HIV-positive and HIV-negative participants with uncomplicated arterial hypertension in rural Tanzania and Lesotho.

The trial is designed for a superiority comparison between the triple drug combination regimen versus control, and a non-inferiority comparison between the dual drug combination regimen versus control. Allocation is 2:1:2 for the dual combination, triple combination, and control arms, respectively, with parallel assignment.

### Control and intervention arms

**Fig. 1:**
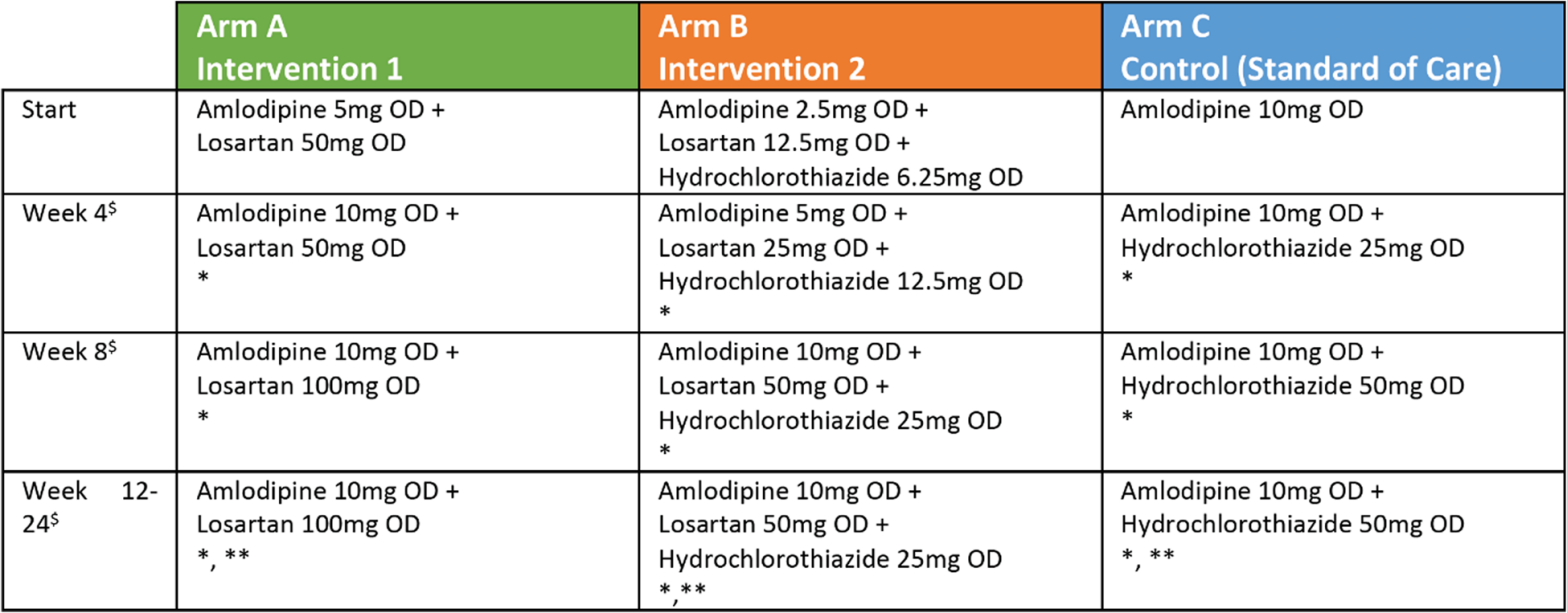
Study interventions and drug dosing according to study arm. OD, once daily. ^$^ Increases in dosages only if target BP is not reached (see above). * In case of orthostatic hypotension or adverse events, medication will be reduced to the prior step—or to half of the initial dosage. ** if regimen shows insufficient effect, individualized adaptation possible according the treating physician

Treatment strategies are shown in Fig. 1. The control arm follows standard of care, i.e., national guidelines of Lesotho and Tanzania, which recommend a CCB or a TZD as first line, and if insufficient, both drugs are combined [10, 20, 38, 39]. For this trial, participants in the control arm start treatment with amlodipine 10 mg. Participants randomized to the dual arm receive a combination of half-dose amlodipine (5 mg) and losartan (50 mg). Participants randomized to the triple arm receive a combination of quarter-dose amlodipine (2.5 mg), hydrochlorothiazide (6.25 mg), and losartan (12.5 mg). The choice of amlodipine, losartan, and hydrochlorothiazide is based on their broad availability and low cost. All of the three drugs are part of the essential drug list by the WHO [40]. Participants in all three arms follow a prespecified dose titration after 4 and 8 weeks if target BP values are not met (Fig. 1).

### Study procedures

#### Screening and eligibility criteria

**Fig. 2:**
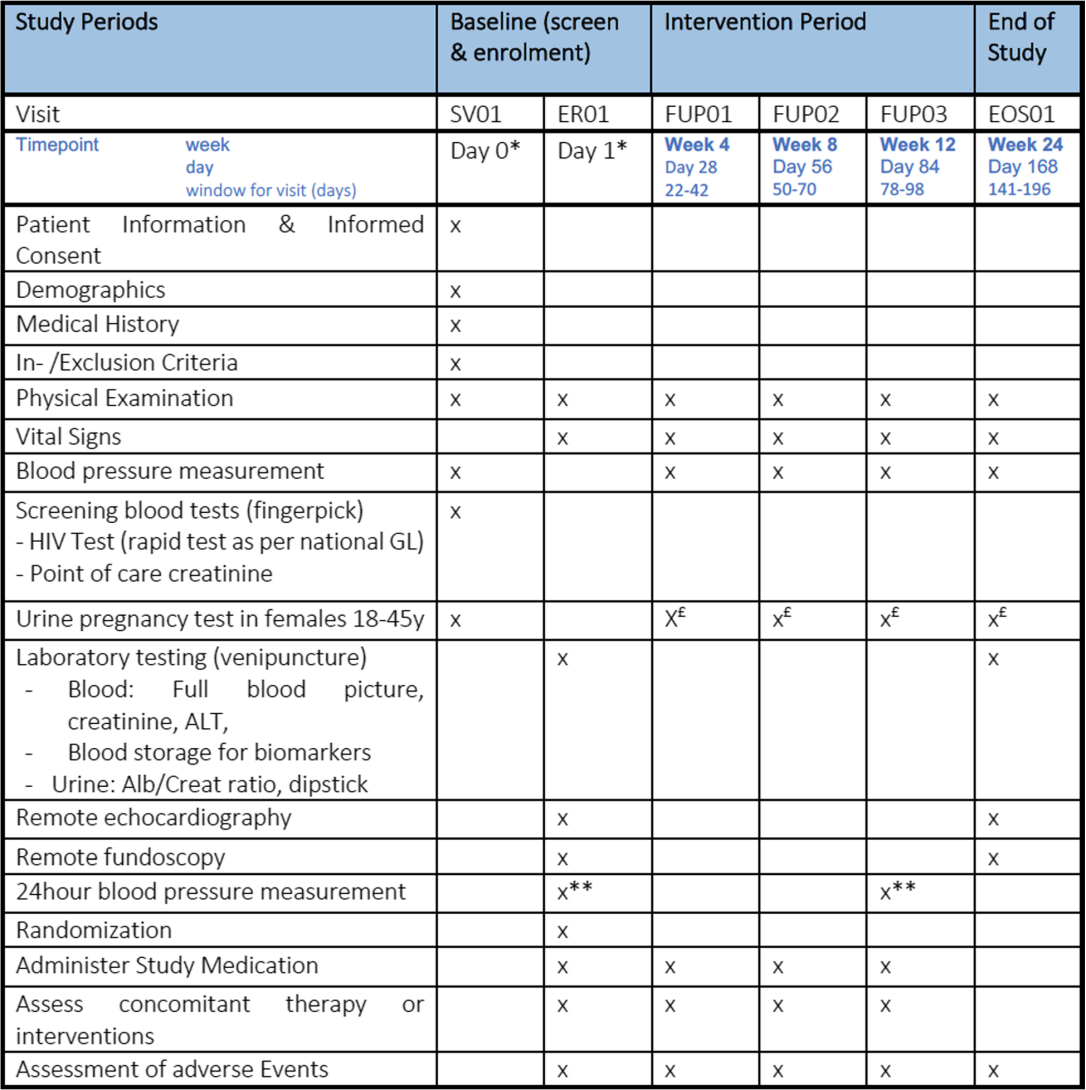
Study visit schedule. SV, screening visit; ER, enrolment; FUP, follow-up; EOS, end of study; ALT, alanine aminotransferase; Alb/Crea, albumin/creatinine; HIV, human immunodeficiency virus. *Day 0 (screening) and day 1 (enrolment) are the same day for participants not enrolled in the 24-h ambulatory BP study, £for all women of reproductive age (18–45 years), ** in 100 participants from Ifakara (nested study)

During routine care at the HIV clinic or the outpatient department, a BP measurement is done, which serves as pre-screening for the study. Individuals with a pre-screening BP ≥ 140/90 mmHg are referred to the study nurse. The study nurse informs the individual about the study, obtains written informed consent, and checks eligibility criteria (Table 1). Screening of participants is a stepwise procedure starting with a questionnaire to ensure absence of acute disease, followed by a standardized office BP measurement (see below). A urine pregnancy test is performed in all women of childbearing age (18–45 years) to exclude pregnancy. From a fingerpick blood sample, an HIV test is done if the participant is not known positive or has not been tested during the last 3 months with a documented result. A point of care creatinine is done to exclude severe renal impairment (creatinine clearance < 30 ml/min) (Fig. 2).

**Table 1:** Inclusion and exclusion criteria for coArtHA trial

Inclusion criteria	Exclusion criteria
- Adults (≥ 18 years of age) - African descent and black ethnicity - Confirmed uncomplicated and currently untreated arterial hypertension* diagnosed at one of the two sites.	- Current hospitalization for any reason - Refusal to do an HIV-test or indeterminate HIV test result - History of cardiovascular event in the last month (angina pain, stroke, myocardial infarction or respective diagnosis by a doctor) - Symptomatic arterial hypertension • Blood pressure ≥ 180/110 mmHg plus headache or chest pain) or acute cardiovascular event (see above) - Acute disease, e.g. • Temperature > 37.5 °C or other signs of acute concomitant infection • Dyspnea/respiratory distress • Acute pain - Clinical signs of hypertension-mediated organ damage, e.g. • Heart failure (bilateral pitting edema, bilateral crackles or pleural effusion, distended jugular veins) • Ischemic heart disease (anginal pain on exertion) • signs of current ischemic/hemorrhagic stroke (hemiparesis, loss of consciousness) - Pregnancy (test required for females 18–45 years of age) - Non-consenting or inability to come for follow-up visits - Creatinine clearance ≤ 30 ml/min by the Chronic Kidney Disease Epidemiology Formula (CKD-EPI) estimation and measurement with a point-of care creatinine from capillary blood

#### Enrolment and randomization

Immediately after screening, enrolment is done with a detailed history and a clinical exam. All information is entered into an electronic questionnaire (MACRO®, Elsevier). By venipuncture 5 ml of blood is withdrawn and sent to the laboratory for full-blood count, serum creatinine, and alanine aminotransferase. Urine is analyzed for albumin-creatinine ratio. A 12-lead-Electrocardiogram (ECG), a focused echocardiography using Lumify® device (Philips), and a retinal picture (iExaminer®, Welch-Allyn) are performed (Fig. 2). The results of these analyses are stored electronically for later interpretation by a cardiologist and ophthalmologist.

Randomization is stratified by site (Lesotho, Tanzania), HIV status (negative, positive), and age (< 65/≥ 65 years), using permuted blocks with varying block sizes. The randomization list was prepared in advance by an independent statistician and is stored securely on a server with restricted access. The allocation is concealed by using opaque, sealed, and labeled envelopes prepared by independent persons based on the randomization list. The envelopes are labeled on the outside with the stratification information and a sequential identification number and contain the randomized allocation and subject identification number. The first five randomizations in each stratum are checked in real-time, and subsequent regular checks are performed to ensure that the randomization sequence is respected. The nurse opens the envelope according the stratification, and the study physician fills in an electronic drug prescription according to the arm. The nurse dispenses the drugs accordingly and provides pre-packed and labeled medication for 1 month to the patient. Handing out of study drugs goes along with clear instructions on intake, adherence, and appointment for the next follow-up visit.

#### Follow-up clinic visit procedures

Follow-up visits are scheduled at 4, 8, 12, and 24 weeks after enrolment. During these visits, the study nurse evaluates adherence to the study drugs, asks for symptoms relating to side effects and other adverse events, and performs standardized BP measurements. In women of childbearing age, a pregnancy test is repeated at every visit. The study doctor examines the participant and prescribes study drugs according to the treatment arm (Fig. 1). Participants who reach the target BP and do not report side effects are prescribed the same medication at weeks 4, 8, and 12. In participants, who do not reach the target BP, the drug prescription is adapted by dosage increase or addition of other drugs as per protocol (Fig. 1). Additional visits can be scheduled if clinically indicated. Participants missing their appointment are tracked within a week of the missed scheduled appointment—first by a phone call, and if the participant is not reachable by tracking with the help of community health workers or a person blinded to the allocation going to the participant’s house.

On the last follow-up visit at 24 weeks, participants undergo again examinations to quantify surrogate markers of end-organ damage (Fig. 2). After successful completion of the study, participants are referred to the local medical team for continued management including further prescription of drugs. Participants do not receive any payment to be part of the study besides compensation for transport expenses caused by additional clinic visits.

#### Standardized blood pressure measurement

For the determination of BP, we use a standard operating procedure based on the European Society of Cardiology/European Society of Hypertension (ESC/ESH) guidelines 2018 [41], which has been used in several recent clinical trials and epidemiological studies [42–44]. In brief, arm circumference is measured to determine the cuff size according to the recommendations of the BP device manufacturer (Omron M6 Comfort [HEM-7321-E] [45]. BP measures are taken in sitting position after 5 min of rest with feet on floor; back supported; no caffeine, exercise, or smoking in the 30 min before measurement; emptied bladder; no talking during measurement; comfortable clothes; and arms supported (e.g., on table). At the screening visit, the reference arm is determined by measuring BP on both arms. The reference arm (with higher BP) is noted and used for all further BP measurements. The BP is calculated as the mean value of the last two out of three consecutive measurements, spaced 1–2 min apart.

#### Endpoints

The primary endpoint is the proportion of participants reaching target BP (≤ 130/80 mmHg in participants aged < 65 years and ≤ 140/90 mmHg in participants aged ≥ 65 years) at 12 weeks. We chose this target BP in line with updated European guidelines and the documented beneficial effect on cardiovascular outcomes [41]. The secondary endpoints are defined in Table 2.

**Table 2:** Secondary endpoints and nested studies

Endpoint	Time point after randomization	Remarks
Proportion of participants reaching a target BP of ≤130/80 mmHg in patients < 65 years of age and ≤ 140/90 mmHg in patients ≥ 65 years of age	At 4, 8, and 24 weeks	Same definition of target BP* as for the primary endpoint at 12 weeks
Change in BP from enrolment	At 4, 8, 12, and 24 weeks	Reduction in mmHg
Proportion of participants with treatment adaptations made to the primary treatment	By 12 weeks	Dose increase or decrease, and/or drug additions
Proportion of participants with a blood pressure decrease of at least 20/10 mmHg	4, 8, 12, and 24 weeks	
Number of treatment adaptations per participant made to the primary treatment	By 12 weeks	Dose increase or decrease, and/or drug additions
Time until target BP is (first) reached	Over 24 weeks	Censoring at last visit for those not observed to reach the target BP*, and for patients who achieve the target BP* any subsequent rebounds will be described but not included in this analysis
Proportion of participants with changes in surrogate markers for hypertension-mediated organ damage (resolving, newly occurring or worsening)	Over 24 weeks	Surrogate markers of organ damage • Kidney impairment: decrease in eGFR (CKD-EPI formula); increase in proteinuria, measured by albumin/creatinine ratio or • Hypertensive heart disease: • Positive Sokolow-Lyon Index (Sokolow-Lyon voltage (SV1 + RV5/V6 ≥ 3.5 mV and/or RaVL ≥ 1.1 mV) on ECG [46, 47]) or • Signs of left ventricular hypertrophy [48] or left atrial remodeling/enlargement assessed by focused echocardiography [49, 50] or • Retinopathy: assessed by retinal picture [51].
Proportion of participants with major cardiovascular endpoints	Over 24 weeks	Major clinical endpoints of mortality, major cardiovascular events such as stroke, myocardial infarction, heart failure, end-stage kidney disease
Proportion of participants lost to follow-up or stopped treatment	Over 24 weeks	
Proportion of participants with at least one grade 3/4 adverse event	Over 24 weeks	Adverse events will be graded according to the CTCAE v5.0, January 2018
Proportion of participants with at least one severe adverse event	Over 24 weeks	
Proportion of participants who were non-adherent to study drugs	Over 12 weeks	< 90% pill count or < 90% of self-reported drug intake
Reasons for non-adherence assessed by pill count and self-report	Over 12 weeks	Descriptive analysis

*BP* blood pressure, *eGFR* estimated glomerular filtration rate, *CKD-EPI* Chronic Kidney Disease Epidemiology Collaboration, *ECG* electrocardiogram, *CTCAE* Common Terminology Criteria for Adverse Events, *HIV* human immunodeficiency virus

*Target BP is defined as ≤ 130/80 mmHg among participants aged < 65 years and ≤ 140/90 mmHg among participants aged ≥ 65 years

#### Sample size calculation

We hypothesize that the proportion of participants reaching the primary endpoint will be higher in the triple combination arm compared to the control arm. Additionally, we hypothesize that the dual combination arm will be non-inferior to the control arm (Table 3). We assumed a response rate in the control arm of 40%, an improvement in the triple combination arm of 15 percentage points (two-sided alpha of 0.05) for the superiority comparison between the triple combination and control arms, and a non-inferiority margin of 10% (one-sided alpha of 0.025) for the non-inferiority comparison between the dual combination and control arms. Based on these assumptions, we calculated a sample size of 431 participants in each of the control and dual combination arms, and 216 participants in the triple combination arm (power of 85% for the non-inferiority comparison and 95% for the superiority comparison). The overall sample size is therefore 1078 participants, with the randomization ratio of 2:1:2 for the dual combination, triple combination, and control arms, respectively. Assuming 15% of participants will become lost-to-follow-up [29] brings the total required sample size to 1268 individuals.

**Table 3:** Assumptions for sample size calculation

	Dual combination Guideline + incremental value of ARB in African patients	Triple combination quarter dose for 3 widely available drugs used	Control WHO standard of care starting with monotherapy
Literature	Reported response in 67% of Africans (response = diastolic blood pressure < 90 mmHg or 10% decrease [10, 52])	Reported response in 83% of patients* (response = blood pressure < 135/85 mmHg [17])	Reported response in 67% of patients in Nigeria (response = blood pressure < 149/90 mmHg [53])
Conservative effect estimation for higher target BP^£^	60%	75%	50%
Conservative effect estimation for lower target BP^$^	40%	55%	40%
Comparison with cited studies	Assumption of a smaller effect due to lower BP target	Effect might be lower (3 drugs instead of 4; no single pill) Effect might be higher as allowance to increase dosage	Effect might be lower due to lower target

*ARB* angiotensin receptor blocker, *BP* blood pressure, *WHO* World Health Organization

^£^140/90 mmHg

^$^130/80 mmHg

#### Data collection and management

Baseline information containing demographics and clinical evaluation are filled into a standardized electronic data management system (MACRO®, Elsevier) using password-protected laptops. Participants are assigned a unique identifier at screening and randomization which is used on all study documentation.

Data are checked by the principal investigator and the data manager to ensure complete and accurate data, with queries raised within the electronic data capture system to clarify inconsistencies and missing data. At each site, a master list linking the participant’s unique identifier to the participant’s details such as name is kept in a locked cupboard. Data will be stored in Swiss Tropical and Public Health Institute (Swiss TPH) servers which are located in Basel, Switzerland, with a defined policy in place for server set-up, maintenance, and security. Data are kept in compliance with local legal requirements, for a minimum of 10 years after completion of the study.

#### Analyses

Analyses and reporting will follow CONSORT guidelines [54–56] and intention-to-treat (ITT) principles, that is including participants as randomized. A flowchart will describe the inclusion and follow-up of participants by study arm. Baseline characteristics will be described by study arm with summary statistics such as median and interquartile range or number and percentage; no formal testing between arms will be performed [57]. Outcomes will be described by arm using summary statistics. The primary outcome, the proportion of participants reaching the target BP within 12 weeks, will be assessed using a logistic regression model, reporting odds ratios and risk differences with standard errors estimated using the delta method [58]. Binary secondary outcomes will be evaluated in the same way. Continuous secondary outcomes will be assessed using linear regression models, reporting mean differences. Time to event outcomes will be assessed using Kaplan-Meier estimation and Cox proportional hazards models. Estimates will be reported with 95% confidence intervals (CI). All models will be adjusted for baseline BP and the stratification factors of site, HIV status, and age [59]. Effect modification of the primary outcome by site and HIV status will be assessed by incorporating an interaction between arm and site or HIV status, respectively, acknowledging that power will be low. Appropriate methods such as multiple imputation will be considered to account for participants with missing outcome data. We will compare each of the intervention arms versus control. For the non-inferiority comparison between the dual combination and control arms, a CI approach will be used. A figure illustrating the CIs and the non-inferiority margin will be presented. Primary analyses for the non-inferiority comparison will be performed on both the ITT and per protocol sets [60]. If the dual combination is found to be non-inferior to the control, then we will assess for superiority using the ITT set. The trial statistician will perform the statistical analyses using Stata (version 15, Stata Corporation, Austin, TX, USA). A full statistical analysis plan will be developed.

#### Nested studies and additional analyses

In a subset of 100 consenting participants (with a separate informed consent) living close to the CDCI in Ifakara, 24-h ambulatory BP and standardized unattended BP measurement will be offered, to assess the proportion of participants with white coat hypertension [29]. In consenting participants, the 24-h BP measurement is started immediately after enrolment, before randomization and study drug dispensing. The device is programmed to take measurements every 20 min between 6:00 and 22:00 and every 30 min between 22:00 and 6:00 [61]. At the end of the 24-h ambulatory BP measurement, an unattended automated office BP measurement is done using a Dräger Infinity Delta® monitor, which is programmed to take five consecutive measurements after 5 min of rest, spaced 1 min apart with a calculation of the mean out of all measurements [61–64]. After completing both 24-h and unattended BP measurements, the participant is randomized and receives study drugs as described above. Both, the 24-h ambulatory BP measurement and the unattended BP measurement are repeated at 12 weeks. Results have no influence on randomization but will help to evaluate unattended office blood pressure as a tool to investigate white coat hypertension in low resource environments, where ambulatory blood pressure measurement is not widely available. Participants are informed of the results at the end of the study.

For the cost-effectiveness analysis, we follow the JAMA guidelines and calculate incremental cost-effectiveness of the three regimens from both a health-systems and a societal perspective [65]. Health systems cost will include total medication cost as well as staff time and a fixed cost for each facility visit, which will be compared to the total health benefits achieved by the three arms [66]. Medical cost will be directly collected at the facility level in the two sites; we will obtain WHO reference prices for the respective drugs and treatments for comparison. For the societal perspective, we will include additional private cost of participants, with a particular focus on out-of-pocket expenditure for visits to facilities (transport, overnight stays) as well as costs for additional medication needed and days of work lost due to sickness [65]. To compute incremental cost-effectiveness ratios, we will use the control arm as our reference case and then compute the additional costs and benefits of the two intervention arms relative to this baseline scenario. Health outcomes will directly be observed over a 24-week period; reduced morbidity will be converted to disability-adjusted life years using the 2013 Global Burden of Disease disability weight estimates [67]. A separate analysis plan will be developed.

### Monitoring and independent data monitoring committee

Monitoring is done by the Quality Management team of the Ifakara Health Institute (IHI) in Tanzania and by the Clinical Operations Unit, Swiss TPH in Lesotho. The study sites are visited by the trial monitoring team for site initiation, during the trial and at study closure. An independent data monitoring committee (IDMC) has been established to monitor the trial for efficacy and safety in accordance with an IDMC charter consisting of five members, including clinical experts from both countries and a statistician. An interim analysis to monitor the trial for efficacy and safety is planned after 50% of the target sample size has completed their primary outcome assessment at 12 weeks, which is expected to be approximately 1 year after the start of the trial. Only IDMC will have access to unblinded efficacy and safety data. Whether further analyses are needed and the timing of such analyses will be determined by the IDMC. Furthermore, the IDMC will recommend that the trial continues, be modified, or be terminated based on their review.

### Safety

All trial drugs have a well-established safety profile. Safety outcomes are assessed by adverse events (AE) and serious adverse events (SAE), which are captured at every visit and are documented at the earliest possible time point. (S) AEs are documented, graded according to the common terminology criteria for adverse events (CTCAE), and reported according to ethics regulations of Tanzania, Lesotho, and Switzerland. The study physician is responsible for management and documentation of all (S)AEs. If a participant develops an AE of grade 2 or higher at the last study visit, he/she remains under observation by the study physicians beyond study termination, until the AE is resolved or stabilized.

## Discussion

Worldwide, around 41 million people die annually from non-communicable diseases (NCDs). Arterial hypertension is the most prevalent risk factor for cardiovascular diseases and claims approximately 7.5 million lives annually [68]. Africa has the highest burden of arterial hypertension with an estimated prevalence of 40% [3]. This adds to the burden of the health care systems which are already overwhelmed with the management of a high number of infectious diseases in the region, including long-term care for patients with HIV [68–71]. Despite the high burden in Africa, there are still few clinical trials evaluating the best treatment for arterial hypertension in sub-Saharan Africa [41].

Since more than two thirds of patients need a combination therapy of antihypertensive medications to reach optimal BP targets [17, 18, 20]—which will be even more so in the light of new tighter targets—the question is less about the optimal first-line drug class but rather the optimal combination and strategy to reach the target in the shortest time frame. A recent randomized controlled trial found that CCB-containing regimens were superior compared to a combination of diuretics and ACE-inhibitors among Africans [29]. Additionally, BP control has not been studied with respect to possible interactions with ART in people living with HIV, likely affecting treatment response [72–75]. This is of particular public health importance since more than 60% of HIV-infected patients worldwide live in sub-Saharan Africa [76].

With the coArtHA trial, we aim to address these gaps by investigating three different regimens of widely available antihypertensive drugs listed in the WHO essential drug list in HIV-positive and HIV-negative participants with uncomplicated arterial hypertension in rural Tanzania and Lesotho. Furthermore, the trial evaluates surrogate markers of end-organ damage such as renal impairment, cardiac function, and ocular manifestations.

We foresee some limitations; firstly, that 24-h BP measurement is not feasible for all participants posing a risk that we miss white coat hypertension in participants living remotely from the facility [77, 78]. We chose a pragmatic approach using a highly standardized, stringent technique of office BP measurement and plan a nested study to compare in a subset of participants the office BP with a 24-h ambulatory BP measurement. Secondly, since this is an open-label study, both participants and study staff are aware of the treatment allocation. However clinical endpoints such as ECG, echocardiography, retinal picture, and 24-h ambulatory BP measurements will be interpreted by clinicians blinded to the study arm.

In summary, the coArtHA trial will inform on the best treatment strategies for uncomplicated arterial hypertension in people living in sub-Saharan Africa. The trial aims to inform future guidelines, to assess hypertension-mediated end-organ damage, and to determine the cost-effectiveness of different arterial hypertension treatment strategies.

### Timeline

The study duration of the study is planned to be 18 months, with a 12-month recruitment period followed by 6 months of follow-up. Recruitment started at Mokhotlong District Hospital on March 06, 2020, and at St. Francis Referral Hospital, Ifakara, on March 24, 2020. Due to national lockdown measures during the SARS-Cov-2 pandemic, recruitment was interrupted in Lesotho from March 29, 2020, and in Tanzania from March 30, 2020. Recruitment was resumed in Lesotho on May 20, 2020, and in Tanzania on June 08, 2020. In case of slow recruitment, it might be extended to nearby hospitals. As of 28.07.2020, the number of participants recruited was 142.

## Data Availability

A minimal verified and anonymized dataset will be made available to a public data repository.

## Abbreviations

ACE: Angiotensin converting enzyme
AE: Adverse events
AIDS: Acquired immunodeficiency syndrome
ARB: Angiotensin receptor blocker
ART: Antiretroviral treatment
BP: Blood pressure
CCB: Calcium channel blocker
CDCI: Chronic Diseases Clinic of Ifakara
CI: Confidence intervals
CKD-EPI: Chronic Kidney Disease Epidemiology Collaboration
CoArtHA: Control Arterial Hypertension in sub-Saharan Africa
CTCAE: Common terminology criteria for adverse events
ECG: Electrocardiogram
eCRF: Electronic case report forms
ESC: European Society of Cardiology
ESH: European Society of Hypertension
HIV: Human immunodeficiency virus
IDMC: Independent data monitoring committee
IHI: Ifakara Health Institute
IHI-IRB: Ifakara Health Institute – Institutional Review Board
ITT: Intention-to-treat
JAMA: Journal of the American Medical Association
KIULARCO: Kilombero and Ulanga Antiretroviral Cohort
NCDs: Non-communicable diseases
NIMR: National Institute for Medical Research
SAE: Serious adverse event
Swiss TPH: Swiss Tropical and Public Health Institute
TMDA: Tanzania Medicines and Medical Devices Authority
TZD: Thiazide diuretic
WHO: World Health Organization
